# Research on the evolutionary game and simulation of multiple subjects’ behavior in the reconstruction of old urban residential communities

**DOI:** 10.1371/journal.pone.0339495

**Published:** 2026-01-02

**Authors:** Yongchang Jiang, Yanjun Dong

**Affiliations:** School of Management, Harbin University of Commerce, Harbin, China; USTC: University of Science and Technology of China, CHINA

## Abstract

The successful execution of the reconstruction of old urban residential communities, a significant project that affects people’s livelihoods, depends on the cooperative efforts of several subjects. However, existing research predominantly focuses on dyadic interactions between governments and residents, neglecting the critical role of third-party enterprises. This study advances the field by developing a tripartite evolutionary game model encompassing the governments, district residents, and third-party enterprises, which we simulate using Matlab and empirically examined based on the case studies. The study’s findings demonstrate that, regardless of the strategies selected by the various participants, the final strategy selections will converge to the stable equilibrium state of (1,1,1). In addition, the primary factors influencing each participant’s strategy selections are the governments incentive mechanism, the cost of renovation for district residents, and the profits and losses of third-party enterprises. These insights provide both theoretical advancements in multi-stakeholder urban governance models and practical guidance for optimizing collaborative dynamics, mitigating social tensions, and improving the overall efficacy of community renewal initiatives.

## Introduction

The reconstruction of old urban residential communities is a crucial component of the organic renewal of cities and a key initiative for the implementation of livelihood projects in China, which have a significant impact on enhancing residents’ quality of life, optimizing the functional layout of cities, and promoting the high-quality development of the economy. According to the National Bureau of Statistics as well as data from the Ministry of Housing and Construction, there are 160,000 old urban residential communities in China that need to be reconstructed, with a reconstruction area of about 4 billion square meters, involving about 42 million households, but the reconstruction task has been completed by only a quarter. In July 2020, the General Office of the State Council issued the Guiding Opinions on Comprehensively Promoting the Reconstruction of Old Urban Neighborhoods. It explicitly places the reconstruction of old urban residential communities on the work agenda of the governments at all levels. In addition, the 2024 government work report also emphasized strengthening the old urban residential communities and the supply of guaranteed housing, enhancing the construction of barrier-free environments and aging-friendly facilities, and creating livable, innovative, and resilient cities. Under the guidance of this goal, localities have combined the actual introduction of relevant supporting measures in their regions. However, it is worth noting that the reconstruction of old urban residential communities, as a prominent people’s livelihood and development project, despite specific achievements and the formation of several experiences that can be replicated, is still faced with problems such as conflict of values, insufficient allocation of resources for public services, and difficulties in financing throughout the entire process of promoting the project.

The reconstruction of old urban residential communities is not only an improvement of the living environment, but also a process of redistribution of benefits. Therefore, under the goal of high-quality development, how to gather the consensus and wisdom of all parties to continuously and steadily promote the reconstruction of old urban residential communities, and realize the benign interaction between government governance, market regulation, and user participation has become an important issue. However, the current research does not take into account the influence of the transformation behavior of multiple subjects and their interaction mechanism on the transformation process, instead relying on the cooperation background of “government-led, market-operated” to carry out the evolutionary game between the two sides. Based on the above background, this study constructs a three-party evolutionary game model from the three main dimensions of the governments’ role, district residents’ demands, and third-party enterprises’ participation, aiming to analyze the decision-making logic of each participant and their possible combinations of strategies in different situations, to identify stable equilibrium strategies that can promote the long-term stable development of the system. At the same time, Matlab 2016b software is used to simulate the evolution process of the behavior of the multiple subjects and the influencing factors, and empirically examined based on the case studies. Finally, countermeasures are proposed based on the simulation results to guide the stakeholders involved in the reconstruction of the old urban residential communities to have a benign interaction, to provide management inspiration for the major problems that need to be solved urgently by the academia and the industry, and to provide strategic guidance for accelerating the high-quality and synergistic development of the economy.

## Literature review

Old urban residential communities refer to residential communities (including single residential buildings) in cities or counties (towns) that were built earlier, are in disrepair and out of control, have inadequate municipal facilities, inadequate community service facilities, and have a high desire among the populace to renovate. With the improvement of people’s living standards and the high-quality development of China’s economy, the issue of old urban residential communities with outdated facilities and incomplete functions is becoming more and more prominent. It is also getting more challenging to meet the needs of residents for a better living environment. These communities are a significant problem that needs to be resolved for the sake of contemporary social progress and historical issues that were left behind during the urban development process. In response to this problem, the reconstruction of old urban residential communities has come into being, which not only involves the consideration of investment and return at the economic level but also profoundly affects the change of social structure, the improvement of the ecological environment, and the inheritance and development of local culture.

As an essential part of urban regeneration, the reconstruction of old urban residential communities has become a hot topic of common concern in recent years for both academia and industry scholars, both domestically and internationally, who have dedicated themselves to investigating more rational and scientific solutions through multi-angle and interdisciplinary research methods, which has led to the accumulation of fruitful academic and practical achievements in the reconstruction concept, reconstruction modes, and benefit distribution of the reconstruction of old urban residential communities. During the evolution of the reconstruction concept, the idea of sustainable development has been deeply rooted in people’s hearts, which has prompted the reconstruction of old urban residential communities from purely pursuing the speed and scale of the physical environment regeneration in the past to a new stage of paying more attention to the people-oriented, comprehensive benefit enhancement [1]. This reconstruction is not only embodied in the improvement of the living conditions of the residents but also involved in the innovation of the community governance mode, the improvement of public service facilities, the protection of the ecological environment, and many other aspects. Especially with the increasingly apparent characteristics of social aging, aging-appropriate reconstruction has become one of the critical concerns of governments at all levels [2,3]. This means that more consideration will be given to their needs during the reconstruction of elderly residential communities, such as adding barrier-free facilities, optimization of medical service support, and other measures to ensure that this group can enjoy a more convenient and comfortable living environment. In addition, scholars have also proposed that the green reconstruction of old urban residential communities can not only effectively promote the achievement of energy conservation and emission reduction goals but also identify fresh possibilities for economic growth [4–7]. Regarding the complex and sensitive issue of benefit distribution, some academics have explored how to balance the relationship between the various stakeholders in upgrading old urban residential communities from the perspective of the distribution of the land value-added proceeds [8]. They investigated profit-sharing coefficients by maximizing the premise of overall interests [9]. Additionally, some academics have used the evolutionary game as an analytical tool and established an optimization model to simulate the interaction between the government and the market, as well as between the residents and the market and the process of their behavioral strategy selection [10,11]. In terms of reconstruction modes, based on the different compositions of the participating bodies, it can be classified into the top-down mode, which the government mainly guides; the market-oriented reconstruction mode, which is participated by enterprises and social organizations; and the community self-management mode, which is committed to the establishment of long-term management mechanisms. In terms of the different composition of the participants, they can be divided into government-led top-down models, market-oriented reconstruction models with the joint participation of enterprises and social organizations, community self-organic reconstruction models that aim to establish a long-term management mechanism, and quasi-property management models of a welfare nature [12–14]. Additionally, the research that is currently available indicates that while Singapore depends primarily on the government to take a top-down approach to transformation and provide funding to encourage residents to actively participate, the United States of America relies on market forces led by non-governmental organizations to transform its old neighborhoods. In contrast, the Japanese government is primarily responsible for the overall transformation of the planning, guidance, and financial support [15,16].

The process of the reconstruction of old urban residential communities is a complex and systematic endeavor that requires consideration of the shared role of the governments, district residents, and third-party enterprises in balancing the interests of various stakeholders. This will help ensure that the transformation process proceeds smoothly. Although scholars have pointed out that under government guidance, models such as Public-Private Partnerships (PPP) and Build-Operate-Transfer (BOT) can encourage third-party enterprises and residents to participate in reconstruction [17], and existing literature has accumulated substantial research on tripartite government-market-resident game frameworks in fields like PPP models and urban renewal [18], current studies still exhibit two limitations regarding the dynamic evolutionary mechanisms of multi-subject collaborative governance in the specific context of reconstructing old urban residential communities: (1) Most literature simplifies governments and the public into a binary gaming framework of “government group vs. investment group” [19], failing to adequately incorporate the impact of residents’ strategic choices as property rights holders on systemic equilibrium; (2) Existing tripartite gaming models predominantly focus on macro-level institutional design, lacking dynamic evolutionary analysis of micro-level parameters such as cost-sharing and resident participation costs (C₂) in reconstructing old urban residential communities. A theory known as evolutionary games examines conflicts of interest between two or more subjects and strategy selection, with the goal of examining how different subjects choose strategies to achieve the balanced growth of interests among several subjects [20]. Building upon existing research on tripartite gaming, this study constructs a tripartite linkage evolutionary game model involving governments, residents, and third-party enterprises, specifically addressing the unique characteristics of reconstructing old urban residential communities (e.g., high proportion of resident self-funding, sensitivity to benefit distribution). Through Matlab simulations analyzing systemic evolutionary trends and local influencing factors, combined with empirical case studies, we propose strategies to balance multi-subject interests. This framework maintains tripartite cooperative stability, establishes a balanced benefit pattern, and provides a context-specific theoretical extension and practical reference for collaborative governance in reconstructing old urban residential communities.

## Construction of the evolutionary game model

### Multi-subject definition

The reconstruction of old urban residential communities is a complex governance project that involves the governments, district residents, and third-party enterprises. As such, there are conflicting and intertwined interests, and it is necessary to coordinate the various demands of various subjects in order to implement public policies, protect housing rights and interests, and acquire market benefits. This will force them to continuously adjust their strategic choices and maximize their own interests.

The governments play the role of a coordinator through policy guidance and financial support in order to accomplish the goal of urban renewal by striking a balance between top-down supervision and horizontal collaboration, with the goals of political (urban renewal), social (credibility of performance), and economic (cost savings and GDP growth) benefits; As the primary holder of property rights, district residents benefit directly from the renovations as well as serving as the oversight body, emphasizing financial restitution, asset appreciation, and the enhancement of living standards. While the third-party enterprises concentrate on profit maximization, depending on government subsidies, community service revenues, and brand premiums to achieve short-term gains and long-term cooperation opportunities, direct beneficiaries and supervisors are more concerned with financial compensation, asset appreciation, and improving living quality. They also need to be involved in decision-making to protect their own rights and interests.

Nonetheless, the three parties’ conflict of interest results from their different objectives. Conflicts exist between the government and third-party enterprises over the importance of social benefits and profit-driven speculation (e.g., jerry-built materials sparked by moral hazard); the government and district residents misalign demands (e.g., participation costs and marginalization of the issue’s rights) due to poor communication; and the district residents and third-party enterprises due to the quality of the transformation (the enterprise compression costs) and the rights and interests of the district residents to protect the benefits of the development of the antagonist’s formation. In essence, these conflicts are a dynamic game between market efficiency, individual rights and interests, and public objectives. To ensure the sustainability of the transformation process, these conflicts must be handled through the balance of authority and responsibility as well as institutional synergy.

### Research assumptions and parameter settings

Based on the evolutionary game theory, this study proposes the following hypotheses for the conflict of interest and game behavior among the governments, district residents, and third-party enterprises:

Assumption 1: Evolutionary game theory points out that individuals in the group through imitation, trial and error and learning to make dynamic adjustments to the strategy, and eventually converge to a stable equilibrium state. Therefore, based on this theory, it is assumed that the governments, district residents, and third-party enterprises are limited rational subjects; each subject constantly adjusts and changes its own behavioral choices, and ultimately tends to a local stable equilibrium, the goal of the tripartite subject decision-making since the maximization of self-interest.

Assumption 2: The government has two strategic options in the reconstruction of old urban residential communities, namely (regulation and non-regulation); the government will guide the behavior of third-party enterprises through rewards or penalties under the supervision of the government. The probability of government regulation is x, and the likelihood of non-regulation is 1-x, the government’s strategy choice is (x,1-x) (x∊ [0,1]). Combining existing studies, the gains/losses of government regulation/non-regulation of the reconstruction of old urban residential communities are set as R_1_ and L_1_, respectively; the cost of government regulation of third-party enterprises is C_1_; the government’s rewards for the supportive behaviors of district residents are E_1_; and the government’s rewards/penalties for positive/negative behaviors of third-party enterprises are E_2_ and F, respectively. While the model simplifies strategic choices into binary options for analytical tractability, we recognize that real-world decisions exist on a continuum (e.g., partial support from residents, conditional participation by enterprises). Subsequent studies will incorporate multi-strategy frameworks to capture nuanced behavioral responses.

Assumption 3: The district residents have two options: they can support or oppose the reconstruction of the old urban residential communities when the costs are lower than the benefits, the district resident chooses to support the reconstruction of old urban residential communities, otherwise they will choose not to support it. The probability of district residents supporting reconstruction is y, and the likelihood of opposing reconstruction is 1-y, the district resident’s strategy choice is (y,1-y) (y∊ [0,1]). The benefits/costs of the district residents in the reconstruction of old urban residential communities are R_2_ and C_2_, respectively, the gains/losses of the district residents in the positive/negative reconstruction of the third-party enterprises are R_3_ and L_2_, respectively, and the additional benefits of the district residents after the reconstruction of old urban residential communities are R_4_.

Assumption 4: The third-party enterprise has two options: positive reconstruction or negative reconstruction; when the third-party enterprise chooses positive reconstruction, it will respond positively to the government’s call; if the third-party enterprise chooses negative reconstruction, it will be affected by its limited rationality and decide to be passive. The probability of the third-party enterprise’s positive reconstruction is z and the probability of negative reconstruction is 1-z, i.e., the third-party enterprise’s strategy choice is (z,1-z) (z∊ [0,1]). The gains gained by the third-party enterprises’ positive/negative participation in the reconstruction of old urban residential communities are R_5_ and R_6_, respectively, the costs paid by the third-party enterprises when they are positively/negatively reconstructed are C_3_ and C_4_, respectively, and the losses incurred by the third-party enterprises when they are negatively reconstructed are L_3_. The parameters of the game of the government, district residents, and third-party enterprises in the reconstruction of old urban residential communities are as set in Table 1:

**Table 1:** Parameter settings and meanings.

Multiple subjects	Parameters	Meanings
Governments	R_1_	Proceeds received when the government regulates the reconstruction of old urban residential communities
	L_1_	Losses when the government fails to regulate the reconstruction of old urban residential communities
	C_1_	Costs of government regulation of third-party businesses
	E_1_	Costs to the government when regulating third-party enterprises
	E_2_	Government incentives when third-party enterprises are positively reconstruction
	F	Government penalties when third-party enterprises are negatively reconstruction
District residents	R_2_	District residents gained in support of the reconstruction of old urban residential communities
	R_3_	District residents receive benefits when third-party enterprises positively reconstruction
	R_4_	Additional benefits to district residents from the reconstruction of old urban residential communities
	C_2_	Costs to district residents in the reconstruction of old urban residential communities
	L_2_	Losses suffered by district residents in negative reconstruction by third-party enterprises
Third-party enterprises	R_5_	Benefits derived from the positive participation of third-party enterprises in the reconstruction of old urban residential communities
	R_6_	Benefits derived from the negative participation of third-party enterprises in the reconstruction of old urban residential communities
	C_3_	Costs when third-party enterprises positively reconstruction
	C_4_	Costs when third-party enterprises negatively reconstruction
	L_3_	Losses when third-party enterprises negatively reconstruction

### Evolutionary game model

Based on the above assumptions and parameters, based on the evolutionary game theory, the benefit matrix of the government, district residents, and third-party enterprises is created using the evolutionary game theory, as indicated in Table 2.

**Table 2:** Benefit matrix.

Game subject strategy		Third-party enterprises positive reconstruction (z)	Third-party enterprises negative reconstruction (1-z)
Governments regulatory reconstruction (x)	District residents	R_1_-C_1_-E_1_-E_2_	R_1_+F-C_1_-E_2_
	Support for reconstruction	R_2_+R_3_+E_2_-C_2_	R_2_+E_2_-C_2_-L_3_
	(y)	R_5_+E_1_-C_3_	R_6_-C_4_-F-L_3_
	District residents	R_1_-C_1_-E_1_	R_1_+F-C_1_
	Don’t support reconstruction	R_4_	R_4_
	(1-y)	R_5_+E_1_-C_3_	R_6_-C_4_-F
Governments do not regulate reconstruction (1-x)	District residents	-L_1_	-L_1_
	Support for reconstruction	R_2_+R_3_-C_2_	R_2_-C_2_-L_2_
	(y)	R_5_-C_3_	R_6_-C_4_-L_3_
	District residents	-L_1_	-L_1_
	Don’t support reconstruction	R_4_	R_4_
	(1-y)	R_5_-C_3_	R_6_-C_4_

According to Table 2, the expected benefits, average benefits, and replication dynamic equations for the strategic behavior of the governments, district residents, and third-party enterprises can be derived:

(1) The expected benefits to government-regulated reconstruction, the expected benefits to non-government-regulated retrofitting, and the average benefits are $U_{11}$, $U_{12}$, and $\overset{―}{U_{X}}$, respectively:

$$U_{11}=yz(R_{1}-C_{1}-E_{1}-E_{2})+y(1-z)(R_{1}+F-C_{1}-E_{2})+(1-y)z(R_{1}-C_{1}-E_{1})+(1-y)(1-z)(R_{1}+F-C_{1})$$ (1)

$$U_{12}=yz(-L_{1})+y(1-z)(-L_{1})+(1-y)z(-L_{1})+(1-y)(1-z)(-L_{1})$$ (2)

$$\overset{―}{Ux}=xU_{11}+(1-x)U_{12}$$ (3)

(2) The expected benefits of district residents supporting the reconstruction, the expected benefits of district residents not supporting the reconstruction, and the average benefits are $U_{21}$, $U_{22}$, and $\overset{―}{U_{y}}$, respectively:

$$U_{21}=xz(R_{2}+R_{3}+E_{2}-C_{2})+x(1-z)(R_{2}+E_{2}-C_{2}-L_{2})+(1-x)z(R_{2}+R_{3}-C_{2})+(1-x)(1-z)(R_{2}-C_{2}-L_{2})$$ (4)

$$U_{22}=xzR_{4}+x(1-z)R_{4}+(1-x)zR_{4}+(1-x)(1-z)R_{4}$$ (5)

$$\overset{―}{U_{y}}=yU_{21}+(1-y)U_{22}$$ (6)

(3) The expected benefits of positive reconstruction by third-party enterprises, the expected benefits of negative reconstruction by third-party enterprises, and the average benefits are $U_{31}$, $U_{32}$, and $\overset{―}{U_{z}}$, respectively respectively:

$$U_{31}=xy(R_{5}+E_{1}-C_{3})+x(1-y)(R_{5}+E_{1}-C_{3})+(1-x)y(R_{5}-C_{3})+(1-x)(1-y)(R_{5}-C_{3})$$ (7)

$$U_{32}=xy(R_{6}-C_{4}-F-L_{3})+x(1-y)(R_{6}-C_{4}-F)+(1-x)y(R_{6}-C_{4}-L_{3})+(1-x)(1-y)(R_{6}-C_{4})$$ (8)

$$\overset{―}{U_{z}}=zU_{31}+(1-z)U_{32}$$ (9)

Therefore, the replicated dynamic equation for the strategy choices of the governments, district residents, and third-party enterprises is:


$$F(x)=\frac{dx}{dt}=x(U_{11}-\overset{―}{U_{x}})=x(1-x)(U_{11}-U_{12})=x(1-x)\left[-yE_{2}-z(E_{1}+F)+R_{1}+F+L_{1}-C_{1}\right]$$ (10)

$$F(y)=\frac{dy}{dt}=y(U_{21}-\overset{―}{U_{y}})=y(1-y)(U_{21}-U_{22})=y(1-y)\left[xE_{2}+z(R_{3}+L_{2})+R_{2}-R_{4}-C_{2}-L_{2}\right]$$ (11)

$$F(z)=\frac{dz}{dt}=z(U_{31}-\overset{―}{U_{z}})=z(1-z)(U_{31}-U_{32})=z(1-z)\left[x(E_{1}+F)+yL_{3}+R_{5}+C_{4}-R_{6}-C_{3}\right]$$ (12)

## Evolutionary game equilibrium analysis

### Stability analysis of the Government

Derivation of the replicated dynamic equation for the government is obtained: $\begin{array}{c} \frac{\partial F(x)}{\partial x}=(1-2x)\left[-yE_{2}-z(E_{1}+F)+R_{1}+F+L_{1}-C_{1}\right] \end{array}$. The stability of the replication dynamics equation shows that there is a stabilizing equilibrium strategy when $F(x)=0,\frac{\partial F(x)}{\partial x}<0$.

**Fig 1:**
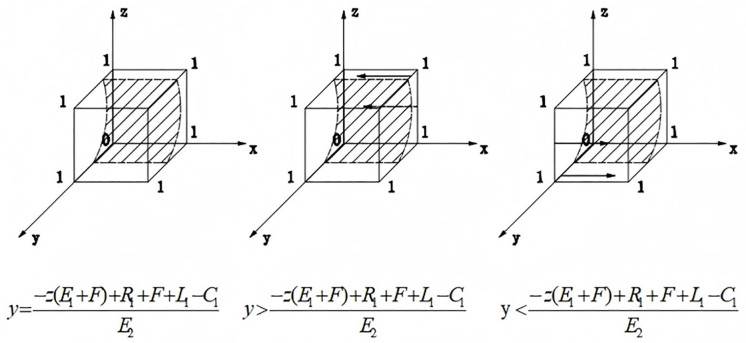
Evolutionary phase diagram of government.

(1) When $y=\frac{-z(E_{1}+F)+R_{1}+F+L_{1}-C_{1}}{E_{2}}$, F(x)≡0, regardless of the value of x, the governments’ strategic choices do not change over time, i.e., the governments’ strategic choices do not change.
(2) When $y\neq \frac{-z(E_{1}+F)+R_{1}+F+L_{1}-C_{1}}{E_{2}}$, let F(x)=0, get x=0 and x=1 two equilibrium points, then need to be divided into two cases for discussion:

When $y>\frac{-z(E_{1}+F)+R_{1}+F+L_{1}-C_{1}}{E_{2}}$, $F^{\prime }(0)<0,F^{\prime }(1)>0$, i.e., x=0 is the stable equilibrium point at which the government does not regulate reconstruction. When $y<\frac{-z(E_{1}+F)+R_{1}+F+L_{1}-C_{1}}{E_{2}}$, $F^{\prime }(0)>0,F^{\prime }(1)<0$, i.e., x = 1 is the stable equilibrium point, at which time the government regulates the reconstruction. The evolutionary phase diagram of the government is shown in Fig 1.

### Stability analysis of district residents

Derivation of the replicated dynamic equation for the district residents is obtained: $\begin{array}{c} \frac{\partial F(y)}{\partial y}=(1-2y)\left[xE_{2}+z(R_{3}+L_{2})+R_{2}-R_{4}-C_{2}-L_{2}\right] \end{array}$. The stability of the replication dynamics equation shows that there is a stabilizing equilibrium strategy when $F(y)=0,\frac{\partial F(y)}{\partial y}<0$.

**Fig 2:**
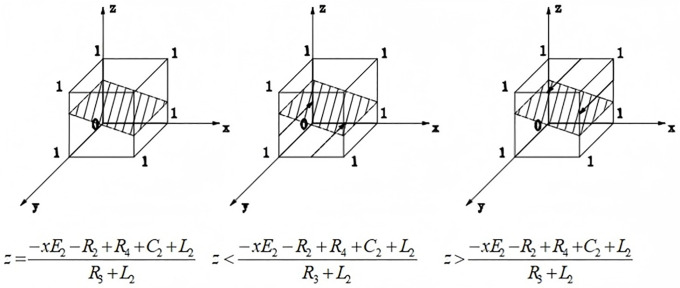
Evolutionary phase diagram of district residents.

(1) When $z=\frac{-xE_{2}-R_{2}+R_{4}+C_{2}+L_{2}}{R_{3}+L_{2}}$, F(y)≡0, regardless of the value of y, the district residents’ strategy choice does not change over time, i.e., the district residents’ strategy choice does not change.
(2) When $z\neq \frac{-xE_{2}-R_{2}+R_{4}+C_{2}+L_{2}}{R_{3}+L_{2}}$, let F(y)=0, get y=0 and y=1 two equilibrium points, then need to be divided into two cases for discussion:

When $z>\frac{-xE_{2}-R_{2}+R_{4}+C_{2}+L_{2}}{R_{3}+L_{2}}$, $F^{\prime }(1)<0,F^{\prime }(0)>0$, i.e., y = 1 is the stable equilibrium point when district residents support the reconstruction. When $z<\frac{-xE_{2}-R_{2}+R_{4}+C_{2}+L_{2}}{R_{3}+L_{2}}$, $F^{\prime }(1)>0,F^{\prime }(0)<0$, i.e., y = 0 is the stable equilibrium point, at this time the district residents oppose the reconstruction. The evolutionary phase diagram of district residents is shown in Fig 2.

### Stability analysis of third-party enterprises

Derivation of the replicated dynamic equation for the third-party enterprises is obtained: $\begin{array}{c} \frac{\partial F(z)}{\partial z}=(1-2z)\left[x(E_{1}+F)+yL_{3}+R_{5}+C_{4}-R_{6}-C_{3}\right] \end{array}$. The stability of the replication dynamics equation shows that there is a stabilizing equilibrium strategy when $F(z)=0,\frac{\partial F(z)}{\partial z}<0$.

(1) When $x=\frac{-yL_{3}+R_{6}+C_{3}-R_{5}-C_{4}}{E_{1}+F}$, F(z)≡0, regardless of the value of z, the strategy choice of the third-party enterprises does not change over time, i.e., the strategy choice of the third-party enterprises does not change.
(2) When $x\neq \frac{-yL_{3}+R_{6}+C_{3}-R_{5}-C_{4}}{E_{1}+F}$, let F(z)=0, get z=0 and z=1 two equilibrium points, then need to be divided into two cases for discussion:

When $x>\frac{-yL_{3}+R_{6}+C_{3}-R_{5}-C_{4}}{E_{1}+F}$, $F^{\prime }(1)<0,F^{\prime }(0)>0$, i.e., z = 1 is the stabilizing equilibrium point, when third-party enterprises reconstruction positively.

**Fig 3:**
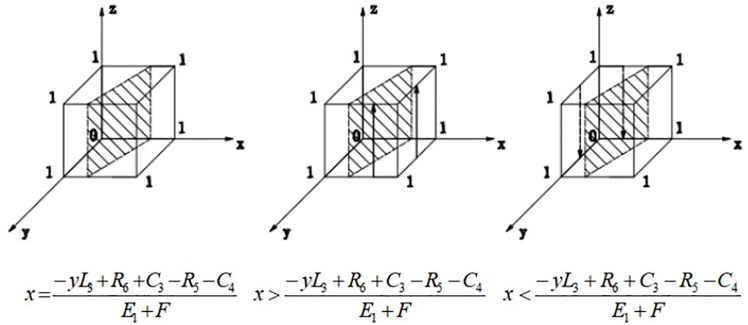
Phase diagram of the evolution of third-party enterprises.

When $x<\frac{-yL_{3}+R_{6}+C_{3}-R_{5}-C_{4}}{E_{1}+F}$, $F^{\prime }(1)>0,F^{\prime }(0)<0$, i.e., z = 0 is the stable equilibrium point, when the third-party enterprises are negatively reconstruction. The evolutionary phase diagram of the third-party enterprises is shown in Fig 3.

### Stability analysis of evolutionary strategies of three-party subjects

**Fig 4:**
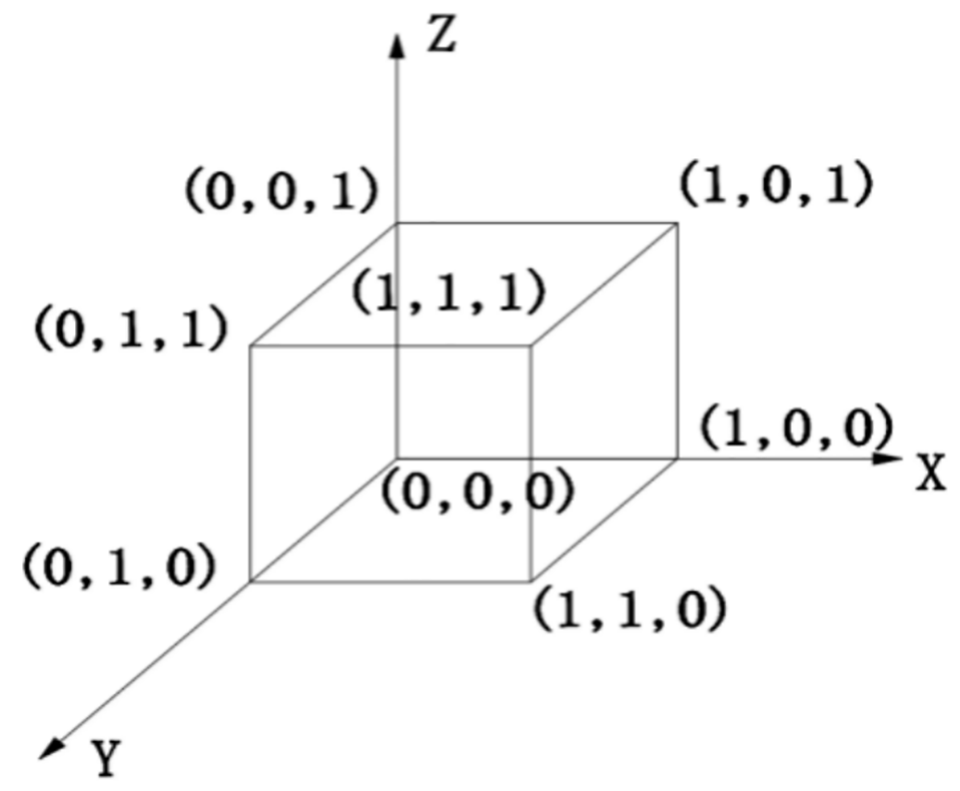
Three-dimensional diagram of the strategy selection interval of the game subject.

From the replicated dynamic equations for the governments, district residents and third-party enterprises, the following eight equilibria exist on R={(x,y,z)|0≤x≤1,0≤y≤1,0≤z≤1}: $A_{1}(0,0,0)$, $A_{2}(1,0,0)$, $A_{3}(0,1,0)$, $A_{4}(0,0,1)$, $A_{5}(1,1,0)$, $A_{6}(1,0,1)$, $A_{7}(0,1,1)$, $A_{8}(1,1,1)$. These 8 points form the boundary of the evolutionary game solution, as shown in Fig 4.

Through the local stability of the Jacobi matrix can be obtained by the stability of the equilibrium point of the evolutionary game, by the governments, district residents and third-party enterprises of the evolutionary game to replicate the dynamic equations can be obtained from their corresponding Jacobi matrix.


$$J=\left[\begin{array}{ccc} {}*20c\frac{\partial F(x)}{\partial x} & \frac{\partial F(x)}{\partial y} & \frac{\partial F(x)}{\partial z}\\ \frac{\partial F(y)}{\partial x} & \frac{\partial F(y)}{\partial y} & \frac{\partial F(y)}{\partial z}\\ \frac{\partial F(z)}{\partial x} & \frac{\partial F(z)}{\partial y} & \frac{\partial F(z)}{\partial z} \end{array}\right]=\left[\begin{array}{ccc} {}*20c(1-2x)\left[\begin{array}{c} -yE_{2}-z(E_{1}+F)\\ +R_{1}+F+L_{1}-C_{1} \end{array}\right] & -x(1-x)E_{2} & -x(1-x)(E_{1}+F)\\ y(1-y)E_{2} & (1-2y)\left[\begin{array}{c} xE_{2}+z(R_{3}+L_{2})\\ +R_{2}-R_{4}-C_{2}-L_{2} \end{array}\right] & y(1-y)(R_{3}+L_{2})\\ z(1-z)(E_{1}+F) & z(1-z)L_{3} & (1-2z)\left[\begin{array}{c} x(E_{1}+F)+yL_{3}\\ +R_{5}+C_{4}-R_{6}-C_{3} \end{array}\right] \end{array}\right]$$

Through analysis of the Jacobi matrix, we may identify whether the local equilibrium point is a stable equilibrium point (ESS). If it fulfills Det(J)>0 and Tr(J)<0, it can be considered a stable equilibrium point with an evolutionary stabilization strategy as its game strategy. Table 3 displays the outcomes of each local equilibrium point after Det(J) and Tr(J) are substituted.

**Table 3:** Results of the stability analysis of the three reconstruction subjects’ evolutionary game.

Equilibrium point	Det(J)	Tr(J)
$A_{1}(0,0,0)$	$\begin{array}{c} (R_{1}+F+L_{1}-C_{1})(R_{2}-R_{4}-C_{2}-L_{2}nonumber\\ \left(R_{5}+C_{4}-R_{6}-C_{3}\right) \end{array}$	$\begin{array}{c} \begin{array}{c} R_{1}+F+L_{1}-C_{1}+R_{2}-R_{4}-C_{2}-L_{2}\\ +R_{5}+C_{4}-R_{6}-C_{3} \end{array} \end{array}$
$A_{2}(1,0,0)$	$\begin{array}{c} -(R_{1}+F+L_{1}-C_{1})(E_{2}+R_{2}-R_{4}-C_{2}-L_{2}nonumber\\ \left(E_{1}+F+R_{5}+C_{4}-R_{6}-C_{3}\right) \end{array}$	$\begin{array}{c} -R_{1}-L_{1}+C_{1}+E_{2}+R_{2}-R_{4}-C_{2}-L_{2}\\ +E_{1}+R_{5}+C_{4}-R_{6}-C_{3} \end{array}$
$A_{3}(0,1,0)$	$\begin{array}{c} (-E_{2}+R_{1}+F+L_{1}-C_{1})(-R_{2}+R_{4}+C_{2}+L_{2}nonumber\\ \left(L_{3}+R_{5}+C_{4}-R_{6}-C_{3}\right) \end{array}$	$\begin{array}{c} -E_{2}+R_{1}+F+L_{1}-C_{1}-R_{2}+R_{4}+C_{2}+L_{2}\\ +L_{3}+R_{5}+C_{4}-R_{6}-C_{3} \end{array}$
$A_{4}(0,0,1)$	$\begin{array}{c} (R_{1}-E_{1}+L_{1}-C_{1})(R_{2}+R_{3}-R_{4}-C_{2}nonumber\\ \left(-R_{5}-C_{4}+R_{6}+C_{3}\right) \end{array}$	$\begin{array}{c} R_{1}-E_{1}+L_{1}-C_{1}+R_{2}+R_{3}-R_{4}-C_{2}\\ -R_{5}-C_{4}+R_{6}+C_{3} \end{array}$
$A_{5}(1,1,0)$	$\begin{array}{c} (-E_{2}+R_{1}+F+L_{1}-C_{1})(E_{2}+R_{2}-R_{4}-C_{2}-L_{2}nonumber\\ \left(E_{1}+F+L_{3}+R_{5}+C_{4}-R_{6}-C_{3}\right) \end{array}$	$\begin{array}{c} -R_{1}-L_{1}+C_{1}-R_{2}+R_{4}+C_{2}+L_{2}\\ E_{1}+L_{3}+R_{5}+C_{4}-R_{6}-C_{3} \end{array}$
$A_{6}(1,0,1)$	$\begin{array}{c} (R_{1}-E_{1}+L_{1}-C_{1})(E_{2}+R_{2}+R_{3}-R_{4}-C_{2}nonumber\\ \left(E_{1}+F+R_{5}+C_{4}-R_{6}-C_{3}\right) \end{array}$	$\begin{array}{c} -R_{1}-L_{1}+C_{1}+E_{2}+R_{2}+R_{3}-R_{4}-C_{2}\\ -F-R_{5}-C_{4}+R_{6}+C_{3} \end{array}$
$A_{7}(0,1,1)$	$\begin{array}{c} (-E_{1}-E_{2}+R_{1}+L_{1}-C_{1})(R_{3}+L_{2}+R_{2}-R_{4}-C_{2}-L_{2}nonumber\\ \left(L_{3}+R_{5}+C_{4}-R_{6}-C_{3}\right) \end{array}$	$\begin{array}{c} -E_{1}-E_{2}+R_{1}+L_{1}-C_{1}-R_{3}-L_{2}-R_{2}+R_{4}+C_{2}+L_{2}\\ -L_{3}-R_{5}-C_{4}+R_{6}+C_{3} \end{array}$
$A_{8}(1,1,1)$	$\begin{array}{c} -(-E_{1}-E_{2}+R_{1}+L_{1}-C_{1})(E_{2}+R_{2}+R_{3}-R_{4}-C_{2}nonumber\\ \left(E_{1}+F+L_{3}+R_{5}+C_{4}-R_{6}-C_{3}\right) \end{array}$	$\begin{array}{c} -R_{1}-L_{1}+C_{1}-R_{2}-R_{3}+R_{4}+C_{2}\\ -F-L_{3}-R_{5}-C_{4}+R_{6}+C_{3} \end{array}$

According to Table 3, the size of and depends on the size of the parameter values. Mathematical derivatives alone can’t determine the equilibrium point nor clarify the formation process of stable equilibrium. Therefore, by assigning values to the parameters and using Matlab 2016b simulation, we can determine the stability of the evolutionary game and make strategy choices.

## Evolutionary game simulation analysis

The previous section used evolutionary game theory to analyze the behavioral choices of the governments, district residents, and third-party enterprises. However, due to the complexity of the parameters, it was difficult to carry out a specific analysis, so the Matlab 2016b software was used to simulate the system and the critical fact, respectively, and final countermeasure suggestions were put forward based on the simulation results.

### Parameter assignment

This study employs the Matlab program to conduct numerical simulation in order to more intuitively describe the model’s evolution path. To ensure the feasibility and rationality of the parameter settings, based on the numerical settings in relevant literature [21–23], combined with the reconstruction of old urban residential communities in China (e.g., high resident self-funding proportion, sensitivity to benefit distribution), and consulting with experts in urban renewal and community governance, the parameters are assigned as follows: R_1_ = 9, R_2_ = 4, R_3_ = 2, R_4_ = 2, R_5_ = 5, R_6_ = 6, C_1_ = 5, C_2_ = 2, C_3_ = 3, C_4_ = 2, L_1_ = 4, L_2_ = 1, L_3_ = 1, E_1_ = 2, E_2_ = 3, F = 3.

### Overall system simulation analysis

**Fig 5:**
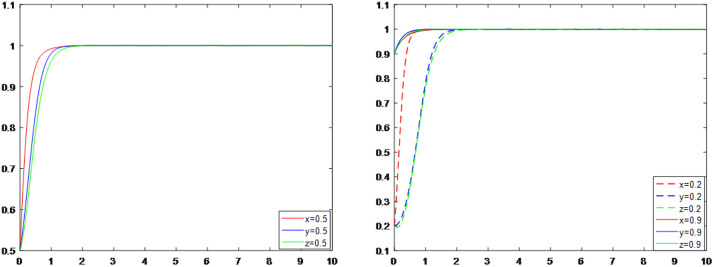
a. Evolutionary trend when the strategy is chosen as the initial value. b. Evolutionary trends as initial values increase or decrease.

Based on the original analysis of the trade-off between interests and the deconstruction of the three-party subjects’ strategic choices, the following characteristics of their synergistic evolution can be revealed in order to further illuminate the internal logic of the synergistic role of multiple subjects and the dynamic evolution of their degree of cooperation: as illustrated in Figs 5a and 5b, the strategic choices of the three subjects eventually converge to 1, meaning that the government regulates the reconstruction, the district residents support it, and the third-party enterprises positively participate in the reconstruction, regardless of whether the subjects’ strategic choices are increasing or decreasing, that is, changing from 0.5 to 0.2 or from 0.5 to 0.9. There are notable temporal differences in inter-subjective strategy interactions, as evidenced by the trend of change. A pilot effect of increased government regulatory intensity led to a gradual increase in community owners’ participation, while third-party enterprises were more cautious in their strategy choices. Their participation increased more quickly when the combination of their two strategies exceeded a threshold value. This strategy dependency relationship shows that corporate decision-making is essentially an adaptive response to the institutional environment and community consensus. Its strategy elasticity coefficient is lower than that of the government and citizens, confirming the risk-averse nature of market participants. The analysis also reveals that a positive feedback loop of “regulatory reinforcement-residents’ response-companies’ follow-up” can occur when the level of government regulation surpasses the critical value, greatly reducing the system’s convergence period.

**Fig 6:**
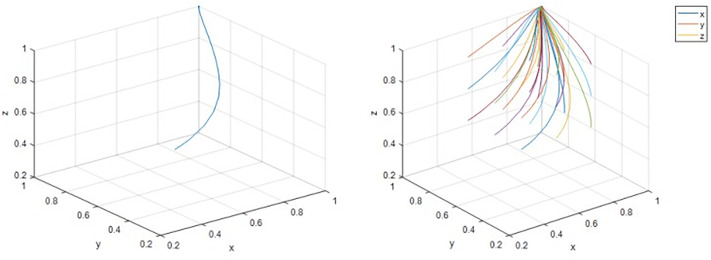
Comprehensive system analysis.

To further explore the strategy selection among multiple subjects, the initial values of the governments, district residents, and third-party enterprises are set to (0.5,0.5,0.5), and the evolution paths of the governments, district residents, and third-party enterprises are shown in Fig 6. This indicates that the degree of cooperation among several subjects evolves according to typical stage characteristics: in the early stage (exploration stage), it is demonstrated through information games and strategy trial and error, and there is a lack of trust and coordination among subjects; in the middle stage (synergistic stage), a two-way commitment mechanism is formed through repeated interactions, and policy tools and community mobilization generate synergy. A multilateral network of reciprocal relationships is formed in the later stage (the symbiosis stage), and mechanisms for risk and benefit sharing encourage cooperation to enter a self-reinforcing channel. At this point, the system achieves a stable equilibrium state of (1,1,1), recognizing the transition from loose coupling to in-depth synergy.

### Simulation analysis of the main influencing factors

#### The effect of government incentives on the evolutionary game.

**Fig 7:**
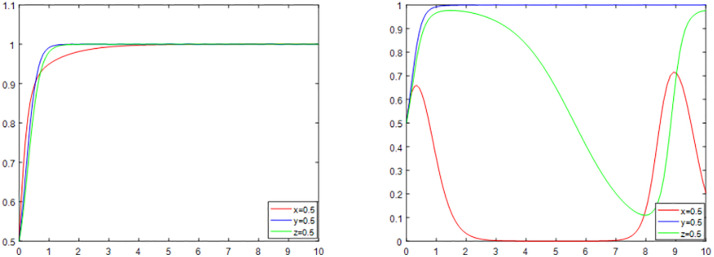
Simulation analysis results under different incentive mechanisms of the government.

To promote the reconstruction of old urban residential communities, the government will provide specific incentives to district residents who support the reconstruction and third-party enterprises. Motivation theory suggests that behavior is a function of outcomes and that motivation and initiative can be stimulated in an individual or organization through appropriate incentives. Suppose other parameters remain unchanged, and the values of E_1_ and E_2_ are changed at the same time. In that case, i.e., the government rewards district residents for their supportive behaviors, and the government rewards third-party enterprises for their positive behaviors, the trend of change is shown in Fig 7. When E_1_ and E_2_ are increasing, according to the theory of reinforcement incentives, the government’s reward mechanism makes the residential communities as well as the third-party enterprises to participate in the renovation of the willingness to increase, as shown in Fig 7, because the rewards, as a kind of positive reinforcement stimulus, increases the likelihood that the behavior will occur again.

However, as the government’s incentive mechanism continues to increase, the probability of the government regulating the reconstruct decreases at this time, and the enterprise may neglect the quality or progress of the reconstruct in order to maximize its own interests. When the government realizes that the loss of not supervising the reconstruct increases, based on the dynamic game relationship between the government and the participants in the game theory, the government will re-increase the probability of supervising, and at this time, the third-party enterprises will choose to actively reconstruct again out of the avoidance of the risk of punishment as well as the consideration of long-term interests.

#### The effect of district residents’ reconstruction costs on the evolutionary game.

**Fig 8:**
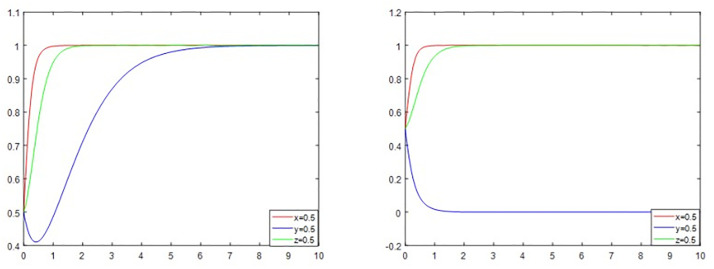
Results of simulation analysis of reconstruction costs for district residents.

With other parameters held constant, the cost to district residents (C_2_) in the reconstruction exhibits a specific trend (Fig 8). At the beginning of the reconstruction, with the increase of the reconstruction cost, the probability of district residents supporting the reconstruction of the declining trend; this tendency can be explained by prospect theory, which holds that people are more sensitive to losses when faced with uncertain decision-making. When district residents perceive escalating retrofit prices, they consider them as a possible loss, which influences their supporting attitude toward reconstructs. With time, the likelihood of district residents supporting the reconstruction of the convergence of 1, which may be due to the community owners by the influence of the government’s incentive policy. Government incentives serve as a positive incentive to alter the expectations and behavioral preferences of district residents, progressively inclining them to favor the reconstruction, according to behavioral economics’ incentive theory.

However, with the gradual increase in the cost of reconstruction, which becomes significantly more than the benefits, the probability of district residents supporting the reconstruction gradually decreases and convergence to 0. This is because, according to the cost-benefit theory and the viewpoint of a rational economic agent, district residents will decide not to support the reconstructing in order to prevent potential losses once they recognize that the high costs involved greatly outweigh the anticipated benefits.

#### Impact of third-party enterprises’ benefits and losses on the evolutionary game.

**Fig 9:**
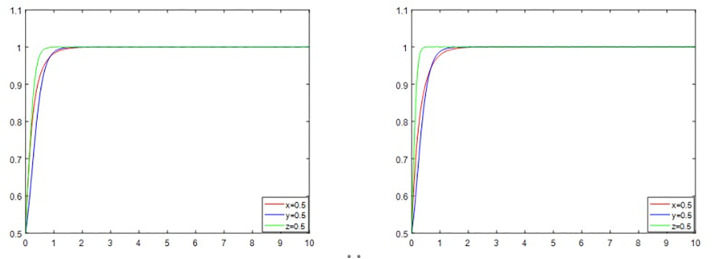
Simulation analysis results when the values of R_5_ and L_3_ are varied separately.

Other parameters remain unchanged, respectively, changing the value of R_5_ and L_3_, that is, the benefits of the third-party enterprises’ positive reconstruction and the loss of the negative reconstruction, and the trend is shown in Fig 9, with the increase of R_5_ and L_3_, the probability of the third-party enterprises’ positive reconstruction shows an increasing trend, and finally converge to 1. The expected utility theory, which holds that third-party enterprises evaluate the value of various options when making decisions, can be used to explain this phenomenon. Businesses are more likely to select positive transformation when the expected utility of positive reconstruction is steadily rising and the gains from positive reconstruction are outpacing the losses from negative reconstruction.

Moreover, with the transparency of information and the improvement of the regulatory mechanism, the perceived behavioral control of enterprises on the behavior of positive transformation is enhanced, and they can more clearly foresee the long-term benefits and sustainable development brought by positive transformation, so in order to pursue the maximization of their own interests, third-party enterprises will choose to positively transform, even if they face short-term difficulties and cost inputs, which ultimately makes the probability of positive transformation continuously The probability of active retrofitting rises and converges to 1. According to the Theory of Planned Behavior, perceptual-behavioral control, attitudes, and subjective norms all have an impact on an enterprise’s behavioral intentions. In this instance, the positive attitude of enterprises toward positive reconstruction is shaped by the high gain of positive reconstruction and the greater loss of negative reconstruction, while subjective normative factors like market pressure and the social environment encourage enterprises to adopt the positive reconstruction behavioral pattern.

### Case validation of simulation results

This study selects the Gongshu District urban renewal project in Hangzhou (2019–2022) for case validation, with comprehensive data obtained from two primary sources: (1) official project documentation from the Hangzhou Municipal Housing Bureau, including feasibility studies, quarterly progress reports, regular satisfaction assessment reports and final acceptance evaluations; and (2) financial statements from three participating enterprises.

Constructed in the 1990s, this area exhibits typical urban renewal challenges including deteriorating building structures, aging public facilities, and insufficient parking spaces. Recognized in 2020 as one of Zhejiang Province’s first provincial-level organic renewal demonstration projects by the Department of Housing and Urban-Rural Development, the initiative creatively adopted a Public-Private Partnership (PPP) model. Through a multifaceted financing mechanism incorporating government subsidies, resident co-financing, and social capital, the project successfully implemented 12 key interventions—ranging from building façade renovation and age-friendly facility retrofitting to smart community system deployment.

According to the 2022 Hangzhou Municipal Old Neighborhood Renovation Satisfaction Assessment Report, after a 24-month renovation cycle, the project achieved significant outcomes: resident satisfaction rose from 62.4 to 87.5 on a standardized 100-point scale, third-party enterprises attained an average return on investment (ROI) of 15.2%, and resident participation in decision-making surged from 61% to 92%. These results contributed to its formal certification by municipal housing authorities in 2022.

In particular, the government of Hangzhou uses a unique incentive approach when it comes to government incentive programs and dynamic regulation. In order to encourage people’ participation, this method includes tax rebates for third-party enterprises (E_2_) and incentives for property owners that assist retrofitting (E_1_). There is initially a lot of regulation, but when companies demonstrate high levels of compliance and active involvement, the frequency of government control gradually decreases. However, when the company receives complaints about the quality of the products, the government will enhance the frequency of inspection through credit demerit systems and fines (L_3_). This results in a closed-loop control mechanism called “incentive-compatible-dynamic punishment.”

In terms of the cost and support of reconstructing by district residents, district residents were initially required to pay 30% of the renovation costs (C_2_), but this percentage was lowered to 15% through the government’s special financial subsidies (R_4_), the distribution of the public maintenance fund, and the sharing of the fund. Following the application of three crucial negotiating techniques: (1) flexible payment plan that permits installment payments over a 24-month period; (2) increased facility allocation (by adding 45 smart parking spaces and 380㎡ communal activity space); and (3) entire process participatory planning, which involved hosting a total of twelve citizens’ council meetings. Over the course of the nine-month implementation period, the support percentage rose to 85%.

In terms of benefits and behavioral drivers for third-party firms, third-party firms obtain 15 years of property management rights (R_5_) through reconstructing, while the contract sets up a stepped liquidated damages clause (L_3_). The initial probability of enterprise participation was 60%, and the final active reconstruct rate rose to 92% due to clear benefits and high default risk. The initial participation rate of third-party enterprises in the reconstruction was 60%, and the high-performance completion rate of 92% was eventually formed after a 6-month risk assessment and strategy adjustment cycle, driven by both clear benefit expectations and rigid risk constraints. According to the available information, the project enters the operation and maintenance stage after the key parameters converge to the stability threshold: the intensity of government supervision remains stable at 0.7, district resident’s comprehensive participation reaches 0.9, and the third-party enterprise’s willingness to renew the contract is 0.95. These empirically observed values demonstrate asymptotic convergence toward the theoretical equilibrium (1,1,1), confirming the model’s dynamic stability in real-world application.

## Discussion

### Main findings

In this paper, an evolutionary game model is constructed using evolutionary game theory to study the governments, district residents, and third-party enterprises as the multiple subjects in reconstructing old urban residential communities. At the same time, they are using Matlab 2016b software to explore the overall evolutionary path and the strategy choices of the three subjects under different parameters. The results show that, regardless of the strategies chosen by the governments, district residents, and third-party enterprises, the ultimate strategy selection will tend to (1,1,1) a stable equilibrium state. At this time, the government supervises the reconstruction of old urban residential communities, district residents choose to support the reconstruction, and the third-party enterprises positively participate in the reconstruction so that the benefits of the three parties are maximized.

Among them, the rewards of the governments (E_1_, E_2_) are the main factor affecting the government’s strategy choice. This pattern implies that a crucial regulatory function in the reconstruction of old urban residential communities is played by policy orientation. Overly high incentives decrease the likelihood of government regulation, thus making the third-party enterprises behave non-participation. As a result, in order to guarantee the anticipation and involvement of all stakeholders, the government must establish the rewards and penalties at a specific ratio while also maintaining a certain balance. This illustrates the need for the government to carefully weigh the interests of all stakeholders when creating policies and to encourage and guide and encourage multiple subjects to jointly participate in the reconstruction of old urban residential communities through reasonable policy design.

The participation cost C_2_ of district residents is the main factor affecting their strategy choice, and their willingness to participate in reconstruction tends to zero when the reconstruction cost is greater than the benefit. This emphasizes that in order to increase district residents’ willingness to support reconstructing, the government should concentrate on lowering their participation costs when creating pertinent policies. This can be done by offering financial subsidies, streamlining the reconstructing program to cut costs, and taking other steps.

The likelihood that third-party enterprises will engage in positive reconstruction tends to rise and finally converge to 1 as the benefit R_5_ of positive reconstruction and the loss L_3_ of negative reconstruction increase. This suggests that when formulating policies, the government should consider how to increase the benefits of positive transformation for third-party enterprises while simultaneously stiffening penalties for negative transformation. This will encourage the enterprises to actively engage in the reconstruction process, allowing them to pursue their own interests while achieving the overall objective of the reconstruction of old urban residential communities.

### Theoretical contributions

This study offers a fresh methodological insight and analytical framework for multi-actor collaborative governance. First, this study fills the theoretical gaps in the existing research on the strategic behaviors of the multiple subjects and the mode of joint governance. In contrast to the existing literature, which primarily focuses on the binary game between the government and the market or between the government and the residents, this study breaks through and constructs a tripartite evolutionary game model of the government, district residents, and third-party enterprises. This model systematically reveals the dynamic equilibrium mechanism of the multiple subjects in the conflict of interests and strategic interactions. Second, by replicating the dynamic equations and Matlab simulation, this study dynamically depicts the stage characteristics and key threshold effects of strategy evolution. This reveals the positive feedback mechanism of “regulatory reinforcement-residents’ response-enterprise followup,” which deepens the application dimension of evolutionary game theory in the reconstruction of old urban residential communities. Most previous studies use static game or equilibrium analysis. This broadens the scope of evolutionary game theory’s application to intricate social governance situations. Furthermore, this paper breaks through the unidimensional limitation of traditional game analysis, analyzes the internal logic of subject behavior from a multidisciplinary perspective, and creatively combines incentive theory, prospect theory, and planned behavior theory. It also offers a more explanatory theoretical framework for comprehending the heterogeneity of behavior of multiple subjects. Finally, through the empirical validation of the instance of Gongshu District in Hangzhou City, the theoretical model is closely linked to the policy practice. This illustrates how the model can be used in actual situations and provides a repeatable analytical framework for future study. This study’s theoretical contribution, in contrast to earlier research, not only expands the range of game subjects and dynamic analysis tools, but it also creates a thorough research chain of “theoretical modeling-simulation-case validation,” providing methodological improvements and helpful references for collaborative governance research in the field of the reconstruction of old urban residential communities.

### Managerial implications

Establish a multi-channel fundraising mechanism to increase funding for reconstruction. it is necessary to address the problem of single-source funding and identify available resources and ways to broaden funding channels. First, the governments should formulate long-term, stable, and forward-looking fiscal policies, allocating a specific percentage of funds annually in the budget for the reconstruction of old urban residential communities. At the same time, the government is actively exploring innovative models for utilizing fiscal funds, such as issuing government bonds, providing fiscal subsidies, and facilitating loans, to offer more flexible financial support for reconstruction projects. Second, revitalize community assets. Specifically, the government can introduce policies to encourage cooperation between enterprises and communities in community planning and construction. For enterprises involved in construction and operation, the government will provide preferential site use and tax breaks to attract their investment in capital and technology, ensuring long-term revenue streams through the collection of management fees.

More importantly, the reconstruction of old urban neighborhoods requires a dynamic funding strategy tailored to the key thresholds identified in the simulation. When resident participation costs (C_2_) exceed benefits (C_2_ + C_4_), governments should prioritize direct subsidies—such as covering 50–70% of renovation expenses—to reduce financial burdens and prevent opposition, particularly for low-income households. For projects where C_2_ remains manageable, a co-financing model can be adopted, with residents contributing 15–30% of costs through flexible payment plans. To align with the profit sensitivity of third-party enterprises, incentives should adapt to participation levels. In early stages (participation probability z < 0.5), long-term contracts (e.g., 15-year management rights) and tax incentives can elevate expected profits (R_5_), while penalties (L_3_) for non-compliance should be stringent (e.g., 150% of delay costs). As participation stabilizes (z ≥ 0.7), rewards can shift to performance-based benchmarks, such as bonuses tied to resident satisfaction scores above 85%. Government regulation should also respond to system dynamics. During low supervision phases (x < 0.6), a combination of high incentives (e.g., 20% tax rebates for compliant firms) and frequent audits ensures rapid engagement. Once the system matures (x > 0.8), oversight can transition to complaint-driven inspections, reducing administrative costs (C_1_) while maintaining accountability. For example, in Hangzhou’s Gongshu District, adaptive subsidies lowered resident costs from 30% to 15% of C_2_, increasing support rates from 60% to 85%. Concurrently, tiered penalties for enterprises raised compliance to 92%. This approach demonstrates how threshold-based policies can optimize outcomes by balancing fiscal sustainability, stakeholder engagement, and project quality.

Enhance the mechanism for expressing interests and cater to the requirements of multiple stakeholders. First, to effectively balance stakeholder interests in the reconstruction of old urban residential communities, policy interventions must be dynamically calibrated to the key thresholds identified in our simulation. When resident participation costs (C₂) approach or exceed their perceived benefits (R₂+R₄), governments should implement tiered legal safeguards-establishing specialized oversight committees to fast-track dispute resolution while mandating participatory budgeting processes that give residents direct input into cost-sharing arrangements. For projects where third-party enterprise engagement (z) falls below 0.5, the legal framework should require transparent bidding processes coupled with performance-based contract clauses that tie at least 30% of enterprise profits (R₅) to resident satisfaction metrics. Second, establishing interest expression channels based on network data terminals is an important way to improve expression efficiency. Leveraging Internet technology, a dedicated online platform for old urban residential community reconstruction should be set up to integrate resources from the governments, district residents, third-party enterprises, and other parties. The platform should be equipped with various functions, such as providing registration and login access points for different stakeholders, allowing all parties to access and express their demands conveniently. The digital expression platform’s design should adapt to these thresholds, automatically triggering enhanced participation mechanisms when key indicators are met. For instance, when resident opposition (1-y) exceeds 20% of the community population, the system could initiate mandatory offline deliberation sessions and activate mediation protocols. The platform’s algorithm should prioritize displaying enterprise proposals that demonstrate clear alignment with both government regulatory standards (x > 0.7) and resident benefit expectations (R₃ > 0.5C₂). Finally, establishing a feedback mechanism for interest demands is key to fostering positive interaction among multiple stakeholders. When various stakeholders express their demands on the platform, government departments, as the primary coordinators and administrators, should promptly categorize and analyze these demands. For reasonable demands, solutions should be formulated quickly, and the results and specific measures should be communicated to the relevant stakeholders via the platform. Feedback mechanisms need particular threshold-sensitive design. When enterprise compliance (z) remains below 0.6 for consecutive quarters, the system should escalate cases to higher-level oversight bodies while automatically adjusting the incentive structure-potentially increasing penalty coefficients (F) by 1.5 times until performance improves. Conversely, when all three stakeholders’ satisfaction scores simultaneously exceed 80%, the system could transition to lighter-touch regulation, focusing on maintaining achieved equilibrium through quarterly check-ins rather than monthly audits. The Gongshu District case demonstrates this approach’s efficacy: their adaptive platform reduced resident opposition from 38% to 12% within 18 months by automatically triggering additional consultation rounds when disagreement thresholds were breached, while enterprise compliance improved from 65% to 88% through dynamically adjusted incentive packages. This threshold-responsive system not only balanced interests but also reduced administrative costs (C₁) by 22% through targeted, rather than blanket, oversight measures.

### Limitations and future research

Admittedly, this paper still has certain limitations under the current research framework, which must be further deepened and expanded in the subsequent research. This study primarily focuses on the game-theoretic interactions among three key stakeholders. While this approach provides a robust analytical framework for understanding the complex dynamics between multiple actors in the reconstruction of old urban residential communities, it inevitably marginalizes other relevant participants. Second, while the binary strategy assumption facilitates equilibrium analysis, it inherently restricts the model’s capacity to represent real-world strategic complexity (e.g., enterprises weighing short-term profit against long-term reputation). Future research will integrate probabilistic strategy mixes—such as residents’ likelihood of supporting reconstruction being influenced by subsidy levels—to more realistically capture strategic dynamics beyond binary choices. Third, future research could refine the model dimensions by incorporating external factors such as social and cultural contexts, policy environment shifts, housing market fluctuations, and group heterogeneity, thereby developing a more comprehensive multi-subject game framework. In addition, the current study may not fully capture the heterogeneity of urban renovation projects across different regions, scales, and socio-economic contexts. Future research will expand the validation framework by incorporating comparative case studies from diverse geographical locations and community types, combined with QCA analysis, to provide a more comprehensive assessment of the model’s effectiveness while generating nuanced, context-specific policy recommendations for various urban renewal scenarios.

## Supporting information

S1 File Relevant code. (DOC)
