# Supplementary material for: Research on the evolutionary game and simulation of multiple subjects’ behavior in the reconstruction of old urban residential communities
Source: PLoS One. 2026 Jan 2;21(1):e0339495. doi: 10.1371/journal.pone.0339495 (PMC12758764; doi:10.1371/journal.pone.0339495)
Supplement: S1 File — (DOC) [file pone.0339495.s001.doc]

%1

function dxdt=gaizao(t,x)

dxdt = [x(1)*(1- x(1))*(-6*x(2)-2*x(3)+9);x(2)*(1-x(2))*(6*x(1)+1*x(3)-2);x(3)*(1-x(3))*(2*x(1)+3*x(2)+3)]

end

%2

for i=0.5

for j=0.5

for m=0.5

[T,X]=ode45('gaizao',[0 20],[i j m]);

figure(1)

grid on

plot(T,X(:,1),'r-','lineWidth',1);

hold on

plot(T,X(:,2),'b--','lineWidth',1);

hold on

plot(T,X(:,3),'g-','lineWidth',1);

hold on

title('演化博弈图');

legend('x','y','z')

end

end

end

%3

i=0.5

j=0.5

m=0.5

[T,Y]=ode45('gaizao',[0 20],[i j m]);

figure(1)

grid on

plot3(Y(:,1),Y(:,2),Y(:,3),'lineWidth',1);

axis([0 1 0 1 0 1])

set(gca,'XTick',[0:0.2:1],'YTick',[0:0.2:1],'ZTick',[0:0.2:1])

xlabel('x');

ylabel('y');

zlabel('z');

title('平衡点8演化博弈图');

hold on

%legend('x','y','z')

%4

for i=0.1:0.2:1

for j=0.1:0.2:1

for m=0.1:0.2:1

set(0,'defaultfigurecolor','w')

[T,Y]=ode45('gaizao',[0 100],[i j m]);

figure(1)

grid on

plot3(Y(:,1),Y(:,2),Y(:,3),'lineWidth',1);

axis([0 1 0 1 0 1])

set(gca,'XTick',[0:0.2:1],'YTick',[0:0.2:1],'ZTick',[0:0.2:1])

xlabel('x');

ylabel('y');

zlabel('z');

title('演化博弈图');

hold on

legend('x','y','z')

end

end

end
